# Disease Ecology, Biodiversity, and the Latitudinal Gradient in Income

**DOI:** 10.1371/journal.pbio.1001456

**Published:** 2012-12-27

**Authors:** Matthew H. Bonds, Andrew P. Dobson, Donald C. Keenan

**Affiliations:** 1 Department of Global Health and Social Medicine, Harvard Medical School, Boston, Massachusetts, United States of America; 2 Department of Ecology and Evolutionary Biology, Princeton University, Princeton, New Jersey, United States of America; 3 THEMA, Université de Cergy-Pontoise, Cergy-Pontoise, France; ISEM, France

## Abstract

While most of the world is thought to be on long-term economic growth paths, more than one-sixth of the world is roughly as poor today as their ancestors were hundreds of years ago. The majority of the extremely poor live in the tropics. The latitudinal gradient in income is highly suggestive of underlying biophysical drivers, of which disease conditions are an especially salient example. However, conclusions have been confounded by the simultaneous causality between income and disease, in addition to potentially spurious relationships. We use a simultaneous equations model to estimate the relative effects of vector-borne and parasitic diseases (VBPDs) and income on each other, controlling for other factors. Our statistical model indicates that VBPDs have systematically affected economic development, evident in contemporary levels of per capita income. The burden of VBDPs is, in turn, determined by underlying ecological conditions. In particular, the model predicts it to rise as biodiversity falls. Through these positive effects on human health, the model thus identifies measurable economic benefits of biodiversity.

## Abstract 2

Vector-borne and parasitic diseases are drivers of the latitudinal gradient in income, and the burden of these diseases is predicted to rise as biodiversity falls.

## Author Summary

While most of the world is thought to be growing economically, more than one-sixth of the world is roughly as poor today as their ancestors were hundreds of years ago. The extremely poor live largely in the tropics. This latitudinal gradient in income suggests that there are biophysical factors, such as the burden of disease, driving the effect. However, measuring the effects of disease on broad economic indicators is confounded by the fact that economic indicators simultaneously influence health. We get around this by using simultaneous equation modeling to estimate the relative effects of disease and income on each other while controlling for other factors. Our model indicates that vector-borne and parasitic diseases (VBPDs) have systematically affected economic development. Importantly, we show that the burden of VBPDs is, in turn, determined by underlying ecological conditions. In particular, the model predicts that the burden of disease will rise as biodiversity falls. The health benefits of biodiversity, therefore, potentially constitute an ecosystem service that can be quantified in terms of income generated.

## Introduction

**Figure 1:**
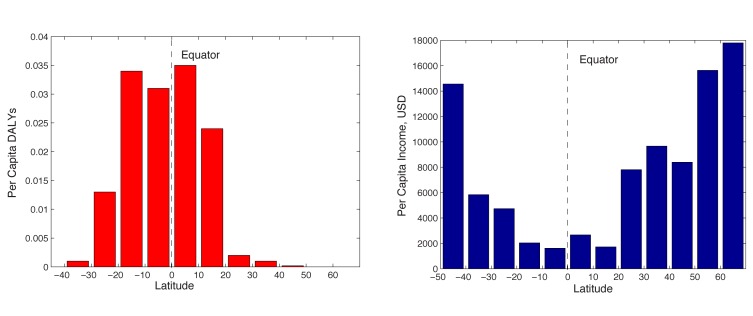
(Left) Per capita DALYs lost to VBPDs along a latitudinal gradient. (Right) Per capita income across latitude is inversely correlated with the burden of VBPDs [1]–[3].

Despite long-term economic growth trajectories for most countries, extreme poverty persists for more than one-sixth of the world. The distribution of wealth and poverty has a clear geographic signature. Along with 93% of the global burden of vector-borne and parasitic diseases (VBPDs), the tropics host 41 of the 48 “least developed countries” and only two of 34 “advanced economies” (Figure 1) [1]–[3].

The latitudinal gradient in income is highly suggestive of underlying biophysical drivers. Latitudinal gradients are found among an extraordinarily wide range of intra- and inter-specific biological processes, from the evolution of animal body size to species diversity, and have served as centerpieces of a number of over-arching paradigms in evolutionary and ecological theory [4]–[11]. These common patterns suggest an opportunity for natural scientists to contribute to a more unified understanding of the role of biological processes in economic development [12]–[15].

Among the many potential biological drivers, the burden of VBPDs stands out as fundamental to explaining geographic distributions of income. VBPDs continue to be among the leading causes of morbidity and mortality of poor populations. Unlike directly transmitted diseases, VBPDs spend much of their life cycle outside of the human host, in other host species or in free-living stages, and are thus especially dependent on external environmental conditions. There is now a consensus among many economists that at least some VBPDS, such as malaria and hookworm, have systematically influenced economic growth [13],[14],[16]–[18].

However, intense debate remains on the relative importance of general disease burden indices on global patterns of wealth and poverty. One side of this debate argues that tropical climates harbor more infectious diseases and offer inferior agricultural conditions, which together influence the overall level of health in the population [13],[14],[16],[19]–[22]. This is thought to directly harm the acquisition of human capital and labor productivity, and increase mortality rates [23]. The corresponding low life expectancies are known to also influence more subtle household allocations of resources, such as reproductive behavior, child-rearing, and long-term private investment.

On the other hand, some have argued that the effect of geography on development has only been through its historical influence on the formation of government and economic institutions [24]–[26]. Under this scenario, geographic constraints—notably, health conditions—have limited the movement of people and foreign investment that would have created the institutions necessary for long-term economic growth. Property rights, for example, did not enjoy constitutional protection in Central Africa because disease conditions prevented foreign settlers from establishing themselves successfully [24]–[26]. Instead of pro-trade institutions, extractive institutions were formed, and then preserved through reinforcing mechanisms over the course of modern history. In this literature too there is implicit agreement that the geography of human health has had significant impacts on economic development [24]–[27]. However, these effects are interpreted as due to the historical consequences of European colonial expansion, and are not considered intrinsically relevant to economic productivity today. Here, we query the validity of these analyses, which assumed that the underlying disease burden influenced the survival of European colonizers but not that of contemporaneous indigenous populations. The ultimate question is whether health effects are actively important today or are only a relic of history.

The distinction of whether the physical environment has systematically impacted economic productivity directly or only indirectly is important for both practical and theoretical reasons. If health is a fundamental ingredient of economic growth, then health care and nutrition would be essential components of macroeconomic strategies for poor countries, and would also be justified targets of foreign economic aid. However, if appropriate economic institutions are the sole significant barriers to economic development, then such aid may have no long-term economic benefits and would only be justified on humanitarian grounds [28].

There are enormous implications for how we understand broad-scale economic processes if they are systematically coupled to biogeographic and ecological phenomena. The literature on the ecology of disease transmission and evolution suggests intrinsically different behavior of infectious and parasitic disease than is typically assumed by economic models, and raises the importance of initial conditions on long-term outcomes [29]–[31]. An important example of the role of ecological processes on shaping human disease burdens is represented in the growing literature on biodiversity and health [32],[33]. Because VPBDs are dependent on other host species, competing parasites, and predators, their abundance may be sensitive to assemblages of other organisms in the ecosystem. Generally, high species densities increase the number of species that prey on disease vectors and free-living parasites. Lyme disease and malaria are but a few examples of diseases that have been documented to increase with the loss of other species in their food webs [34]–[36]. However, there is also evidence that diversity of plants, mammals, and birds are broadly correlated with diversity of human diseases [37]. This hypothesis is further supported by the fact that biodiversity and human disease burdens are also correlated along a latitudinal gradient.

The possibility that these economic-ecological systems are coupled creates challenges for measuring causal pathways and points to the importance of scientific knowledge for informing statistical analysis. Here, we rely on the latitudinal gradient in income as a unifying framework to pursue a question of significance to the ecology, public health, and economic development literature: what are the relative effects of the burden of VBPDs and per capita income on each other? In pursuit of this question, we develop a statistical model that addresses an independently important question in disease ecology: what is the general impact of species diversity on the burden of VBPDs? To measure these relationships, we estimate simultaneous equations of per capita income and the burden of VBPDs, controlling for a range of factors. We find that the latitudinal gradient in income is explained by both the quality of institutions and the burden of VBPDs. The burden of VBPDs is, in turn, determined by underlying ecological conditions. In particular, it is predicted to rise as biodiversity falls.

### Model Development

The primary challenge for understanding relationships between the ecology of human health and global patterns of economic development through statistical analysis of country-level indicators is the problem of endogeneity [38]: economic activity is hypothesized to be both a cause and a consequence of health. Simple ordinary least squares regression analysis would therefore produce biased estimates.

Endogeneity problems are addressed in econometrics through structural equation methods that rely on instrumental variables (IVs) in multi-stage regressions (for details on IVs see Methods) [39]. IVs must be “relevant” and “excludable”—i.e., correlated with an endogenous explanatory variable of interest but not independently correlated with the dependent variable. There have been a number of studies that have attempted to measure the economic impacts of disease through IV methods [16],[23],[24],[26],[40],[41]. All such studies are limited by a general tradeoff between using broad-based health indicators (such as life expectancy or disability-adjusted life years [DALYs]), which are likely to have the most significant economic impacts, and identifying plausible instruments that are not independently correlated with income. While narrower health indicators, such as specific infectious diseases, are easier to instrument for, their effects on aggregate outcomes are more difficult to measure. As a result, conclusions from this literature have been challenged based on questions of the legitimacy of the instruments [42],[43].

In light of these issues, we focus on the per capita burden of VBPDs as our health indicator; this has several advantages. First, VBPDs have been especially implicated in impacting economic growth. While many directly transmitted diseases, such as measles and influenza, are known to have had significant impacts on global mortality rates, their systematic relationship to economic growth over long time scales is less direct. Their high rates of transmission and short infectious periods are associated with rapid acquisition of host immunity, which often lasts a lifetime. Many directly transmitted diseases are also known as “crowd diseases” and tend to be associated with modern economically driven urbanization, and are less dependent on external environmental conditions. In contrast, VBPDs, such as malaria, leishmaniasis, schistosomiasis, ascariasis, and hookworm, are more often associated with longer infectious periods, diminished immunity, and serial reinfection. They spend much of their life cycle outside of the human host in other animal hosts or free-living stages, and are thus especially dependent on external environmental conditions [44],[45]. While etiologically varied, their common ecological properties provide a basis for instrumentation.

**Figure 2:**
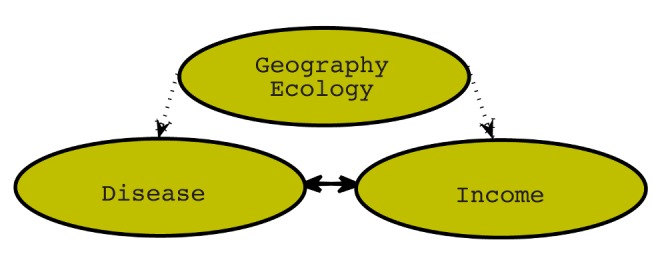
Schematic of the statistical model. The burden of VBPDs and income are estimated simultaneously, with exogenous geographic and ecological variables used as IVs. The IVs for disease are islands and species richness. These are strongly correlated with the disease burden but not independently correlated with income, and therefore can be used to make inference on the effect of disease on income.

We accordingly use a structural equation modeling approach that estimates two simultaneous equations for income and the disease burden, using relevant geographic and ecological variables as IVs [46]. A schematic of the analysis is presented in Figure 2, which corresponds to the following structural equations:
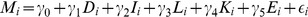
(1)

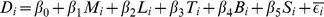
(2)where *M* represents the natural log of per capita income, and the subscript *i* corresponds to the country; *D* represents the natural log of per capita DALYs lost to the following VBPDs: malaria, trypanosomiasis, Chagas disease, schistosomiasis, leishmaniasis, lymphatic filariasis, onchocerciasis, dengue, Japanese encephalitis, ascariasis, trichuriasis, and hookworm [1]; and *I* is a composite index of six World Bank Governance Indicators (WGI): voice and accountability, political stability and absence of violence, government effectiveness, regulatory quality, rule of law, and corruption [47]. The variable, *L*, represents distance in latitude from the equator; *T* is a dummy variable for whether the country is located in the tropics; *K* is a dummy variable for whether the country is landlocked; E is the natural log of the per capita value of oil, natural gas, and coal production; *B* is a biodiversity index based on the species richness of plants, birds, and mammals; *S* is a dummy variable for whether the country is an island; and 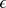 and 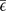 are error terms. All variables are for the year 2002 unless otherwise noted. The model structure is discussed in detail in the Methods section, which also presents analysis of a wide range of alternative model specifications. More details on the variables can be found in Table S1 (Text S1).

## Results

### Results for Income

**Figure 3:**
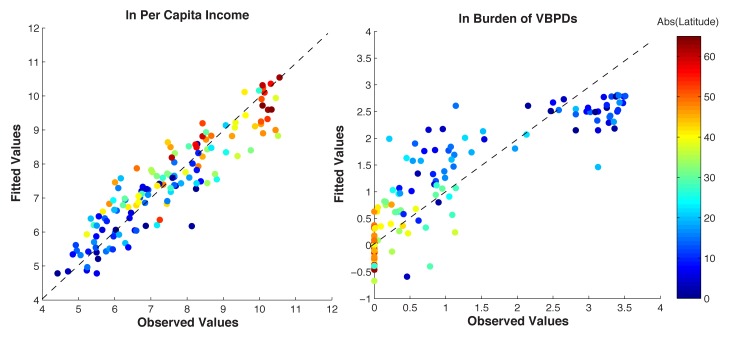
The observed values of income (ln, per capita) and disease (ln, per capita) for each country are presented along with their corresponding fitted values from the models, which fit the data well. The dashed line represents the “perfect fit”; *R* ^2^ = 0.84 and 0.76. The color represents the absolute value of the latitude.

Table 1 presents the results of our analysis, which tells a coherent story of the relationship between the geography of VBPDs and income (*R* ^2^ = 0.84). The coefficient estimate of the impact of VBPDs on income, γ*_1_*, is −0.40, and is significant at the 1% level. This suggests that the average tropical country, with a logged per capita burden of VBPDs of 1.99, would more than double their per capita income if their disease burden were reduced to that of an average temperate country of 0.19. The effect of VBPD burden on income is also found to be statistically significant in all other supplementary analyses (Methods). Other statistically significant explanatory variables for income are the quality of institutions (γ*_2_* = 0.38), the value of primary energy production (γ*_5_* = 0.12), and landlocked status (γ*_4_* = −0.54). These results broadly echo general conclusions from the literature [13],[48]. The fitted values of the model are presented along with the observed values in Figure 3 (left panel).

**Table 1:** Results of simultaneous equations model.

Dependent Variable: Per Capita Income		Dependent Variable: Per Capita Burden of VBPDs	
Independent Variables	Parameter Estimate (Standard Error)	Independent Variables	Parameter Estimate (Standard Error)
**Disease** ln **^IV^**	**−0.40 (0.10)** ***	**Income** ln **^IV^**	**−0.16 (0.09)** *
**Latitude** a	0.24 (1.01)	**Latitude** a	**−2.99 (0.81)** ***
**Landlocked**	**−0.54 (0.21)** ***	**Biodiversity** a	**−0.29 (0.05)** ***
**Energy** ^In^	**0.12 (0.03)** ***	**Island**	**−0.63 (0.30)** ***
**Institutions^IV^**	**0.38 (0.14)** ***	**Tropics**	**0.96 (0.21)** ***
**Constant**	**8.10 (0.25)** ***	**Constant**	**3.33 (0.50)** ***
***R^2^***	0.84	***R^2^***	0.76
**Under-identification tests**		**Under-identification tests**	
**Shea's partial ** ***R*** **^2^, VBPDs:**	0.33	**First-stage ** ***F*** **-test (** ***p*** **-value)**	(0.00)
**Shea's partial ** ***R*** **^2^, inst:**	0.06	**Partial ** ***R*** **^2^, lngdp:**	0.24
**Over-identifying restriction test** J **(** ***p*** **-value)**	(0.89)	**Over-identifying restriction test** J **(** ***p*** **-value)**	(0.73)
**IV Moran's ** ***I*** ** (** ***p*** **-value)**	(0.58)	**IV Moran's ** ***I*** ** (** ***p*** **-value)**	(0.11)

Columns 2 and 4 represent parameter estimates for the income and disease equations, which correspond to equations (1 and 2) in the text. The corresponding independent variables are listed in columns 1 and 3. The income, disease, and energy variables are natural logged. The estimated effect of disease on income is −0.40. This suggests that the average tropical country with a logged per capita burden of VBPDs of 1.99 would more than double their per capita income if their disease burden were reduced to that of an average temperate country of 0.19. The estimated effect of biodiversity on disease is −0.29. Thus, if the biodiversity index of a country like Indonesia (index = 663) were to lose 15% of its biodiversity (falling by 100), the burden of VBPDs would be expected to rise by about 30%. Robust standard errors are presented in parentheses below their corresponding coefficient estimates. First stage *F*-test is used in the second model (column 3) because there is only one endogenous variable (income). Because the first model (column 1) has multiple endogenous variables (disease and institutions), we use Shea's Partial *R* ^2^ as an indicator of the strength of correlation of the IVs [75]. Bold indicates significance at the 10% level (n = 139).

J Based on Hansen's J statistic.

ln Natural log.

a Units×10^−2^ units.

* p≤0.10.

*** p≤0.01.

### Results for Disease

**Figure 4:**
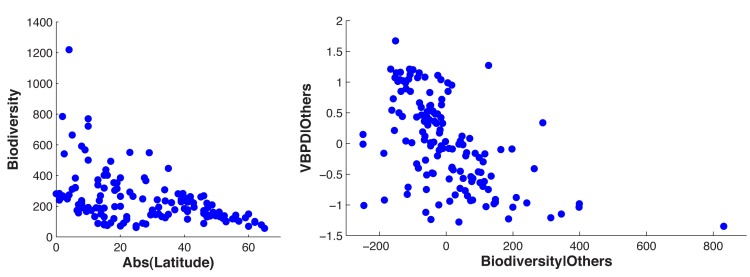
(Left) Each dot represents a country. The biodiversity index is a composite index of species densities of plants, birds, and mammals, based on species area curves for every country; it is strongly correlated with the absolute value of latitude. (Right) Partial correlation plot of the relationship between biodiversity and the burden of VBPDs. All analyses indicate that biodiversity is associated with lower disease burdens after controlling for other factors.

The model for the VBPD burden also appears to be well-specified, with an *R* ^2^ of 0.75 and statistical significance at the 1% level for most of the explanatory variables. Consistent with the literature, the VBPD burden falls with income (β*_1_* = −0.16), absolute latitude (β*_2_* = −2.99), island status (β*_5_* = −0.63), and rises discretely in the tropics (*β*_3_ = 0.96). The coefficient estimate for biodiversity (β_4_ = −0.29) is significant at the 1% level and suggests that if a country with a relatively high biodiversity index of 663 (such as Indonesia), were to lose 15% of its biodiversity, the burden of VBPDs would be expected to rise by about 30%. Figure 3 (right panel) presents the VBPD burdens along with the fitted values. Figure 4 (left panel) presents the biodiversity index along the latitudinal gradient, and Figure 4 (right panel) depicts the partial correlation of biodiversity and the burden of VBPDs.

## Discussion

As far back as Darwin and Wallace's theory of evolution, which was inspired by Malthus' *An Essay on the Principle of Population*, natural scientists have systematically borrowed theoretical approaches from economics. In the modern era, economic tools such as game theory, optimization theory, and time series analysis, have significantly contributed to our understanding of a range of biological systems, from the evolution of pathogen virulence and animal behavior, to the analysis of population dynamics and ecosystem structure [49]–[55]. However, with a few exceptions [56],[57], integration in the reverse direction (from biology to economics) has lagged behind, leaving many open questions on broad-based biological determinants of economic growth.

The economic conditions of the extremely poor are, indeed, largely due to biological processes, which are manifest in health status [58],[59]. Infectious and parasitic diseases effectively “steal” host resources for their own survival and transmission [60],[61]. These within-host processes at the individual level scale up to global patterns of poverty and disease, and are evident along a latitudinal gradient. What drives these patterns?

There are significant differences between the respective approaches of economics and the natural sciences to understanding the importance of geographic and latitudinal variation. Correlated with latitude is a seemingly endless list of biophysical and socioeconomic phenomena, from soil quality and biodiversity to per capita income and religious diversity. Understanding the latitudinal gradient in biodiversity, for example, is one of many unifying questions in the search for underlying principles of biological organization. Scientists have thus addressed the problem with a correspondingly wide range of approaches and scales of analysis, from population genetics and kinetic theory to population, community, and ecosystem ecology [6]–[10],[62]. The result has been a number of competing paradigms as well as some important consensuses.

The latitudinal gradient in income, in contrast, has not been widely used to explore underlying principles in economics, and does not generally serve as a basis for integration with the natural and physical sciences. One of the most influential explanations in the economics literature is that it is merely an historical artifact, due to the process of colonial expansion from Europe [24]–[27].

Methodologically, one challenge to understanding the relationship between geography, health, and economic development is a lack of scientifically based IVs. For example, [24] used settler mortality rates as an IV for institutions, relying on the assumption that they influenced the formation of institutions but are independent of indigenous health conditions. This finding contradicts basic knowledge in microbiology and epidemiology. Vector-borne diseases, such as malaria, continue to be among the dominant causes of morbidity and mortality of tropical populations, just as they were of colonial settlers; partial immunity is acquired among those (foreign or indigenous) who are able to survive repeated infections [63],[64].

The analysis presented here is based on an opposing hypothesis: VBPDs, while influenced by socioeconomic factors, are also determined by independent ecological processes, thus explaining their geographic signature. Disease conditions have, in turn, persistently influenced economic productivity. Our statistical model is derived from these conceptual differences and accordingly estimates income and the burden of VBPDs simultaneously. We find that the burden of VBPDs has had a substantial and statistically significant impact on per capita income after controlling for other factors. This result stands for a wide range of model specifications.

Among the ecological variables that are found to influence the burden of VBPDs, biodiversity is notable. There is an emerging literature on the relationship between biodiversity and human health, which emphasizes that VBPDs are part of broader ecosystems, and their prevalences are dependent on densities of natural predators, competitors, and other host species [32],[33]. However, understanding broader aggregate relationships have been confounded by three important considerations: (1) general biodiversity indices and disease burdens are positively correlated along a latitudinal gradient [30],[37]; (2) biodiversity and poverty are highly correlated [65]; and (3) the relationship between ecosystem structure and the disease burden may be highly variable over time and space, depending on the specific diseases and specific ecological assemblages [32]. Because of these different factors, a general theory of the effect of biodiversity on VBPDs does not exist. After accounting for the effects of income, geography, and other relevant confounders, we find that biodiversity is predicted to lower burdens of VBPDs. Given the inherent underlying complexity, a fuller understanding requires more detailed studies of these relationships across disease types and ecozones.

The policy implications of these results are straightforward: (1) health conditions have influenced the ability of economies to grow over the long-term, as indicated in differences in contemporary levels of per capita income, and (2) well-functioning, diverse, ecosystems can serve public health interests. The health benefits of biodiversity therefore constitute an ecosystem service that can be quantified in terms of income generated. The theoretical implications may be equally important: economic development is coupled to ecological processes. Such integrated approaches between economics and the natural sciences are therefore necessary for explaining economic heterogeneity around the world and for identifying policies that can lead to sustainable global health and economic development.

## Methods

### Simple Model

Table 1 presents the results of two simultaneous equations estimated from a two-step IV method. For a better understanding of the data and methods, here we first heuristically present a simple example of our statistical model, which is used as a foundation from which we systematically build in control variables. The primary goal of this study is to measure the simultaneous effects of the burden of VBPDs and the distribution of income on each other. In the process of controlling for confounders we address a secondary objective, which is to measure the effect of biodiversity on disease. For heuristic purposes, we begin with a regression model of per capita income as the dependent variable and the burden of VBPDs as an explanatory variable. This approach is guided by a couple of basic statistical considerations, such as avoiding omitted variable bias and simultaneity bias.

Omitted variable bias occurs if the burden of VBPDs is correlated with other variables that are not included in the regression model but are themselves correlated with per capita income. It can be addressed by including the appropriate independent variables into the analysis, the choice of which is guided by theory and previous empirical work. In our preliminary analysis, we control for latitude, which is the most conspicuous variable that is correlated with VBPDs and also may be related to economic activity through other indirect mechanisms.

Simultaneity bias occurs when the explanatory variable is itself a function of the dependent variable. This is a serious issue in our study because poverty is known to be an underlying cause of disease. The standard approach to overcoming simultaneity bias in the econometrics literature is through the use of IVs in a structural equation model [66]. The basic requirements for the IVs are (1) they are correlated with the endogenous explanatory variable (“relevance”) and; (2) they are uncorrelated with the error term (“excludability”) (see *Assumptions and Limitations* in Text S1 for more discussion of IV methods).

Identifying IVs for the burden of VBPD presents an opportunity for disease ecology to inform our understanding of the role of health on economic development. Two IVs for VBPDs that we test in this preliminary analysis are island status and biodiversity. Island status is a natural choice for an IV because: (1) ecological theory tells us that islands should generally have lower disease burdens due to lower rates of immigration/transmission and higher rates of extinction/eradication [35],[67]; and (2) island status is not independently important for economic growth in ways unaccounted for in the model. The characteristics of islands that could have economic relevance is their size and access to ports. Because we do not have complete data for many small islands, the island countries that we include cover a wide range of sizes, locations, and histories (discussed in more detail in *Assumptions and Limitations* in Text S1). We account for port access with a dummy variable for landlocked countries in subsequent models. These properties of the IVs are discussed in more detail in the section, *Assumptions and Limitations of Instrumental Variables* in Text S1.

Biodiversity, however, is a potentially more controversial choice for an IV because the literature on the relationship between biodiversity and health is ambiguous. On the one hand, biologically diverse ecosystems are thought to regulate populations of parasites and vectors through predation, competition, and dilution, putting downward pressure on human disease [32],[33],[35]. On the other hand, species richness has been shown to be correlated with diversity of human pathogens, potentially increasing the burden of disease [37]. The first-stage regression is used to generate fitted values of VBPDs based on the IVs and all other exogenous variables. The first stage regression in this example is:
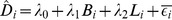
(3)where 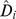 represents the natural log of the per capita burden of VBPDs for country *i*; *B* is an index of the species richness of plants, mammals, and birds (see Table S1 for details); *L* is the absolute value of the latitude; and 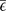 is an error term.

**Figure 5:**
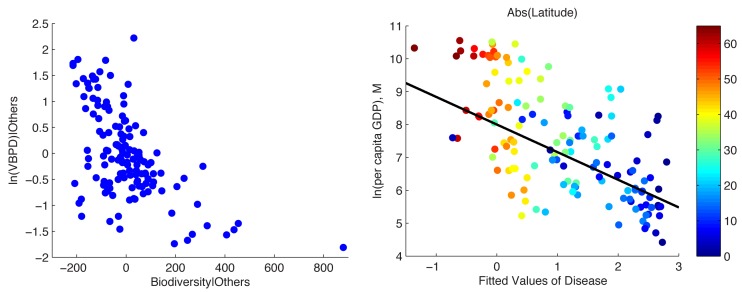
(Left) Partial correlation of biodiversity and the burden of VBPDs estimated from equation (3). (Right) Relationship between per capita income and fitted value of VBPDs, 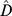, estimated from equation (3).

Column *a* in Table 2 presents the parameter estimates of equation (3). Column b presents results where islands are also included as IVs. Both island status (*p* = 0.00) and biodiversity (*p* = 0.00) are negative and highly statistically significant correlates of the burden of VBPDs. This is further confirmed by a simple *F*-test (in the case of both IVs, we test their joint significance) (*p* = 0.00), such that they easily satisfy the “relevance” criterion [68]. Note that the parameter estimates for biodiversity (−0.34) and islands (−0.71) in these simple first-stage regressions are very similar to the parameter estimates for the full model presented in Table 1 (−0.29 and −0.63, respectively). Figure 5 (left panel) presents the partial correlation of biodiversity and income that corresponds to the results presented in Column *b* of Table 2.

**Table 2:** First-stage regression. Dependent variable: Disease (VPBDs).

Independent Variable	Parameter Estimate (Standard Error)	
	a	b
**Latitude** a	**−6.31 (0.39)** ***	**−6.38 (0.38)** ***
**Biodiversity** a	**−0.33 (0.05)** ***	**−0.34 (0.04)** ***
**Island**	—	**−0.71 (0.23)** ***
**Constant**	**3.59 (0.24)** ***	**3.68 (0.18)** ***
***R^2^***	0.66	0.68
**Partial ** ***R^2^***	0.32	0.36
**First stage ** ***F*** **-statistic (** ***p*** **-value)**	(0.00)	(0.00)

Parameter estimates for first-stage regressions that include biodiversity (columns a and b) and islands (column b) as IVs. Standard errors are presented in parentheses next to their corresponding parameter estimates. Bold indicates significance at the 10% level. *n* = 139.

a Units×10^−2^.

*** p≤0.01.

The second-stage regression is an estimation of the income equation. To overcome simultaneity bias, we substitute the disease independent variable with fitted values of disease from the first-stage regression:
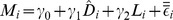
(4)where *M_i_* represents the natural log of per capita income of country *i*, and 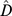 is the fitted value of disease. Note that the IVs for disease (biodiversity and islands) must be excluded from this second-stage regression (otherwise the model is not “identified”). The results of the second-stage regression are presented in Table 3, and the regression line between disease and income that corresponds to Table 3 (column *b*) is presented in Figure 5 (right panel).

**Table 3:** Second-stage regression. Dependent variable: Per capita income.

Independent Variable	Parameter Estimate (Standard Error)	
	a	b
**Latitude** a	**1.67 (0.72)** ***	**1.54 (0.71)** **
**Disease^IV^**	**−0.76 (0.11)** ***	**−0.79 (0.10)** ***
**Constant**	**7.82 (0.27)** ***	**7.88 (0.27)** ***
***R^2^***	0.52	0.52
**Over-identifying restriction test** J **(** ***p*** **-value)**	—	(0.23)

Parameter estimates for second-stage regressions that include biodiversity (columns a and b) and islands (column b) as IVs for disease. Standard errors are presented in parentheses next to their corresponding parameter estimates. Bold indicates significance at the 10% level. *n* = 139.

J Based on Hansen's J statistic.

a Units×10^−2^.

** p≤0.05.

*** p≤0.01.

Testing the excludability criterion is not possible in models with only one IV. However, because the second specification has more IVs than endogenous explanatory variables (it is “over-identified”), we test the over-identifying restriction (Hansen's J). We find no indication that the IVs are correlated with the error term (*p* = 0.23) [69] (for more details see the *Assumptions and Limitations of Instrumental Variables* in Text S1). Despite the simplicity of equation (4), the regression has a relatively high goodness of fit (*R^2^* = 0.52), and is highly consistent with the results from the complete analysis presented in Table 1. Specifically, VBPDs are correlated with lower income, and biodiversity is correlated with lower burdens of VBPDs. Our goal now is to test the robustness of these results through a more rigorous analysis that includes a fuller range of statistical considerations.

### Simple System of Equations

While equation (3) is an appropriate first-stage estimation of disease for the purposes of estimating a second-stage regression of income, it is not complete for our purposes. Because we hypothesize that income and disease influence each other, the most appropriate statistical approach is to simultaneously estimate equations for both variables. Consider the following second-stage equations of interest:
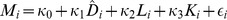
(5)

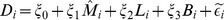
(6)
Equations (5 and 6) represent the simplest possible set of simultaneous equations of income and disease that account for latitude, are “just-identified” (i.e., one IV per endogenous explanatory variable), and can therefore be estimated empirically. They each consist of one IV, which is, by definition, an exogenous explanatory variable in one equation that is excluded from the other equation (for details, see *Assumptions and Limitations* in Text S1). Landlocked status, *K*, is a common control variable in economics because a lack of ports is a major barrier to trade. However, being landlocked is an irrelevant factor for disease transmission and it is thus qualified as an IV for income; biodiversity, *B*, is the IV for disease. The fitted values, 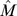 and 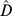, are generated from first-stage regressions: 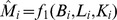 and 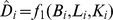.

Equations (5 and 6) are estimated via two-step generalized method of moments [66],[69] with Stata 12. The results are presented in columns 1a and 1b of Tables 4 and 5, respectively. A first-stage *F*-test indicates that landlocked status is a relevant instrument in this simple specification (*p* = 0.00).

**Table 4:** Results. Two-step GMM estimates of simultaneous equations. Dependent variable: Income.

Independent Variables	A. Dependent Variable: Income							
	1a	2a	3a	4a	5a	6a	7a	8a
**Disease** InIV	**^lnIV^−0.65 (0.11)** ***	**−0.66 (0.11)** ***	**−0.62 (0.11)** ***	**−0.38 (0.17)** **	**−0.40 (0.09)** ***	**−0.37 (0.10)** ***	**−0.30 (0.09)** ***	**−0.52 (0.12)** ***
**Latitude** a	**2.38** *** **(0.65)**	**2.32** *** **(0.64)**	**2.36** *** **(0.65)**	0.08 (1.54)	0.24 (1.01)	−0.26 (0.94)	−0.01 (0.01)	−0.11 (1.12)
**Landlocked**	**−1.09 (0.22)** ***	**−1.08 (0.22)** ***	**−0.95 (0.21)** ***	**−0.51 (0.30)** ***	**−0.54 (0.21)** ***	**−0.49 (0.20)** ***	−0.28 (0.20)	**−0.39 (0.17)** **
**Energy^ln^**	—	—	**0.09 (0.04)** **	**0.12 (0.03)** ***	**0.12 (0.03)** ***	**0.12 (0.02)** ***	**0.13 (0.03)** ***	**0.12 (0.03)** ***
**Institutions** IV	—	—	—	0.41 (0.27)	**0.38 (0.14)** ***	**0.41 (0.13)** ***	**0.63 (0.11)** ***	**0.37 (0.17)** **
**Ethnolinguistic fractionalization**	—	—	—	—	—	**−0.44 (0.25)** ***	—	—
**Spatially lagged income** IV a	—	—	—	—	—	—	**0.03 (0.01)** ***	—
**Asia**	—	—	—	—	—	—	—	**−0.70 (0.42)** *
**Southern Asia**	—	—	—	—	—	—	—	**−0.54 (0.20)** ***
**Constant**	**7.77 (0.25)** ***	**7.79 (0.25)** ***	**7.52 (0.29)** ***	**7.81 (0.29)** ***	**7.79 (0.26)** ***	**8.10 (0.25)** ***	**8.03 (0.29)** ***	**8.08 (0.23)** ***
***R^2^***	0.60	0.60	0.62	0.84	0.84	0.84	0.81	0.86
**Under-identification tests**						—	—	—
**First stage ** ***F*** **-test (** ***p*** **-value):**	(0.00)	(0.00)	(0.00)	—	—	—	—	—
**(Shea's) partial ** ***R*** **^2^, VBPDs:**	0.31	0.34	0.34	0.10	0.33	0.34	0.40	0.20
**(Shea's) partial ** ***R*** **^2^, Inst:**	—	—	—	0.02	0.06	0.06	0.09	0.05
**Over-identifying restriction test** J **(** ***p*** **-value)**	—	(0.50)	(0.26)	—	(0.89)	(0.91)	(0.05)	(0.55)
**IV Moran's ** ***I*** ** (** ***p*** **-value)**	(0.25)	(0.24)	(0.28)	(0.61)	(0.58)	(0.67)	(0.80)	(0.75)

From left to right, the number of control variables, which are listed on the left, increases in a stepwise fashion. The IVs for disease are variables that are in the disease equation (listed in the corresponding columns in Table 5) but not in the income equation here. Robust standard errors are presented in parentheses next to their corresponding coefficient estimates. First-stage *F*-test indicates strength of IVs if there is only one endogenous variable (disease). If there are multiple endogenous variables (disease, institutions, and spatially lagged income), Shea's partial *R* ^2^ indicates strength of IVs [75]. Bold indicates significance at the 10% level. *n* = 139.

IV Variable is instrumented.

J Based on Hansen's J statistic.

a Units×10^−2^.

* p≤0.10.

** p≤0.05.

*** p≤0.01.

**Table 5:** Results. Two-step GMM estimates of simultaneous equations. Dependent variable: Disease.

Independent Variables	B. Dependent Variable: Disease							
	1b	2b	3b	4b	5b	6b	7b	8b
**Income** lnIV	**−0.21 (0.10)** **	**−0.16 (0.10)** *	**−0.19 (0.09)** **	**−0.19 (0.09)** **	**−0.16 (0.09)** *	**−0.22 (0.09)** **	**−0.11 (0.07)** *	**−0.17 (0.07)** **
**Latitude** a	**−4.96** *** **(0.76)**	**−5.30 (0.78)** ***	**−0.05 (0.76)** ***	**−0.05 (0.76)** ***	**−2.99 (0.81)** ***	**−2.22 (0.78)** ***	**−2.07 (0.60)** ***	−0.33 (0.67)
**Biodiversity** a	**−0.28** *** **(0.05)**	**−0.30 (0.05)** ***	**−0.29 (0.05)** ***	**−0.29** ***	**−0.29 (0.05)** ***	**−0.29 (0.05)** ***	**−0.22 (0.05)** ***	**−0.09 (0.03)** ***
**Island**	—	**−0.54 (0.32)** *	**−0.56 (0.32)** *	**−0.56 (0.32)** *	**−0.63 (0.30)** **	**−0.62 (0.30)** **	−0.37 (0.25)	**−0.23 (0.15)** ***
**Tropics**	—	—	—	—	**0.96 (0.21)** ***	**1.11 (0.20)** ***	**0.87 (0.19)** ***	**1.00 (0.21)** ***
**Spatially lagged disease** IV a	—	—	—	—	—	—	**0.87 (0.16)** ***	—
**Africa**	—	—	—	—	—	—	—	**1.32 (0.18)** ***
**Central America**	—	—	—	—	—	—	—	**−0.36 (0.12)** ***
**Constant**	**4.65 (0.50)** ***	**4.50 (0.50)** ***	**4.64 (0.48)** ***	**4.64 (0.48)** ***	**3.33 (0.50)** ***	**3.52 (0.51)** ***	**2.35 (0.44)** ***	**1.79 (0.44)** ***
***R^2^***	0.72	0.72	0.73	0.73	0.76	0.76	0.80	0.87
**Under-identification tests**								
**First stage ** ***F*** **-test (** ***p*** **-value)**	(0.00)	(0.00)	(0.00)	(0.00)	(0.00)	(0.00)	—	(0.00)
**(Shea's) partial R^2^, lngdp:**	0.20	0.18	0.24	0.24	0.24	(0.28)	(0.30)	0.27
**Over-identifying restriction test** J **(** ***p*** **-value)**	—	—	(0.40)	(0.40)	(0.73)	(0.03)	(0.03)	(0.03)
**IV Moran's I (** ***p*** **-value)**	(0.07)	(0.08)	(0.08)	(0.08)	(0.11)	(0.13)	(0.17)	(0.54)

From left to right, the number of control variables increases in a stepwise fashion. The IVs for income are the variables that are in the income equation (listed in the corresponding columns in Table 4) but not in the disease equation here. Robust standard errors are presented in parentheses next to their corresponding coefficient estimates. First-stage *F*-test indicates strength of IVs if there is only one endogenous variable (income). If there are multiple endogenous variables (income and spatially lagged disease), Shea's Partial *R* ^2^ indicates strength of IVs [75]. The institutions variable is not included as an IV for income and therefore 4b and 3b are identical. Bold indicates significance at the 10% level. *n* = 139.

IV Variable is instrumented.

J Based on Hansen's J statistic.

a Units×10^−2^.

* p≤0.10.

** p≤0.05.

*** p≤0.01.

### Full System of Equations

Equations (5 and 6) represent a system of equations that are sufficient to estimate the effects of the disease burden and income on each other. As in the simpler regression results presented in Tables 2 and 3, the burden of disease predicts lower income, and biodiversity predicts lower burden of disease. In order to test the robustness of these results, we introduce a fuller range of control variables in a stepwise fashion. There are two criteria that we used in selecting these variables: (1) they have been found in the literature to be determinants of the dependent variable; and (2) they are expected to be exogenous to this system (in particular, they are not determined by income or disease; for more details, see *Assumptions and Limitations* in Text S1).

As mentioned above, one of the primary hypotheses of interest is that the latitudinal gradient in income is partly due to disease ecology. The most prominent competing hypothesis is that it is instead due only to economic institutions. We therefore control for the quality of institutions via a composite index of World Bank Governance Indicators (WGI), similar to other studies (Table S1; Text S1). Because institutions, like disease, are thought to be influenced by income, we also instrument for institutions. Previous studies have used settler mortality rates as IVs for institutions, based on the premise that these mortality rates directly influenced colonial expansion, but are not independently correlated with income today [24],[26],[70]. However, we do not use settler mortality for two reasons: (1) we consider it a direct indicator of disease conditions, which we hypothesize to influence income today (these studies did not separately control for general disease burdens); and (2) there is no data on settler mortality rates for most of the countries in our dataset (only for countries that were colonized). Instead, invoking the same premise as these earlier studies, we allow the IVs for disease to also serve as IVs for institutions. First-stage regression results indicate that the IVs for disease are also statistically significant predictors of institutions (*p* = 0.05; Table S3). Though under-identification tests indicate that the instruments are relatively weak, our inferences are unaffected whether or not institutions is included as a control variable, and whether or not it is instrumented for (these different variations are not presented here).

For income, we consider two more potential IVs: ethnolinguistic fractionalization, *F*, and primary energy production, *E* (for details, see Table S1). Ethnolinguistic fractionalization is a natural consideration because it is considered to be a barrier to trade, a potential cause of civil strife, and is accordingly a common IV in global economic studies [70]. However, over-identification restriction tests indicate that ethnolinguistic fractionalization is strongly correlated with the error term and therefore does not meet the criteria for an IV (Table 4, column 6b); this is highly consistent with recent studies by [71],[72] that the disease burden may itself influence human “assortative sociality” and thereby drive patterns of human diversity. On the other hand, the value of primary energy production (oil, natural gas, and coal) is a useful control variable because it is an exogenous source of revenue for economies. For the disease equation, we add a dummy variable for tropical countries, *T*, because there is overwhelming evidence that many VBPDs thrive in tropical conditions due to metabolic and ecologic reasons [73]. We do not, however, include tropics as a control variable in the income equation because preliminary analyses indicated that tropics are not statistically significant predictors of income, after controlling for other variables (i.e., latitude, disease, and institutions) (*p* = 0.90), but is collinear with institutions. Thus tropical conditions also serves as an IV for disease.

Tables 4 and 5 present the results of eight different specifications of the simultaneous equations estimated by two-step generalized method of moments in Stata 12 (details of the variables are in Table S1). Each of these specifications has been tested for identification (i.e., the strength of the IVs), spatial autocorrelation, and over-identifying restrictions wherever possible. The IV Moran's *I* test measures spatial-autocorrelation in the residuals. Statistically significant spatial-autocorrelation was not found in any of the estimates of the income equation (*p*-values ranged from 0.24 to 0.80), but were found in four of the eight estimates of the disease equation (*p*-values ranged from 0.07 to 0.54). Such spatial autocorrelation in the residuals tends to vanish when additional variables (i.e., that are geographically determined) are controlled for [74]. However, the addition of more IVs increases the possibility of violating the excludability criterion, indicated by the over-identifying restriction test. The last three model specifications suffer from this problem (*p*-values for over-identifying restriction test are less than 0.1 in columns 6b, 7b, and 8b, indicating that the IVs are correlated with the error term). Despite these considerations, the parameters are very consistent across all models. The best overall specification is presented in columns 5a and 5b, which has *R^2^*s of 0.84 and 0.76, is well-identified with strong instruments and no statistically significant spatial autocorrelation. This system is represented by the following reduced-form equations that correspond to structural equations (1 and 2):
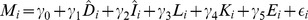
(7)

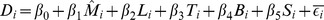
(8)The first stage regressions for the estimation of the income equation (7) are:
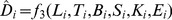
(9)

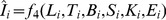
(10)
Table S3 presents the outcomes of these first stage regressions. The identification criteria are easily satisfied. Island status and biodiversity are both significant negative predictors of the disease burden in both simple and more complex models. The first stage regression for the estimation of the disease equation (8) is:
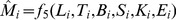
(11)which is presented in Table S4. The identification criteria are easily satisfied here as well. The landlocked and energy variables are especially effective predictors of income. The estimated effect of biodiversity on disease, and of disease on income, were statistically significant for all model specifications.

## Supporting Information

Table S1 **Variable definitions and sources.** Details on variables definitions and data sources.
(DOCX)

Table S2 **Analysis of tropical (a) and sub-tropical (b) countries.** The parameter estimates are presented in columns a, b, and c. The corresponding independent variables are listed to their left. Robust standard errors are presented in parentheses below their corresponding parameter estimates. The lower sample size (and lower variability) among tropical countries results in few statistically significant estimates for the income equation. Nevertheless, the parameter estimates for the impact of disease on income is very similar across groups. **^IV^**Instrumented; ^ln^natural log; ^§^units×10^−2^ units; ***significant at the 1% level; **significant at the 5% level; *significant at the 10% level. **^§^**units×10^−2^.
(DOCX)

Table S3 **First-stage results in the estimation of income** **equation (1)** **.** Columns 2 and 3 represent parameter estimates for equations (9) and (10) respectively. The corresponding independent variables are listed on the left. Standard errors are presented in parentheses below their corresponding coefficient estimates; *n* = 139. ***Significant at the 1% level; **significant at the 5% level; *significant at the 10% level; ^§^units×10^−2^ units.
(DOCX)

Table S4 **First-stage results in the estimation of disease** **equation (2)** **.** The right columns represent parameter estimates for equation (11). The corresponding independent variables are listed on the left. Standard errors are presented in parentheses below their corresponding coefficient estimates; *n* = 139. ***Significant at the 1% level; **significant at the 5% level; *significant at the 10% level; ^§^units×10^−2^ units.
(DOCX)

Text S1 **Supplementary information: variable definitions and data sources; assumptions and limitations of the analysis; supplementary analyses.**
(DOCX)

## Abbreviations

DALY: disability-adjusted life year
IV: instrumental variable
VBPD: vector-borne and parasitic disease
