# Supplementary material for: Disease Ecology, Biodiversity, and the Latitudinal Gradient in Income
Source: PLoS Biol. 2012 Dec 27;10(12):e1001456. doi: 10.1371/journal.pbio.1001456 (PMC3531233; doi:10.1371/journal.pbio.1001456)
Supplement: Table S1 — Variable definitions and sources. Details on variables definitions and data sources. (DOCX) [file pbio.1001456.s001.docx]

| **Table S1. Variable Definitions and Sources** | |
| --- | --- |
| **Variable** | **Information** |
| *M,* *Per capita* income | Year: 2002. Data Source: [1]. |
| *D*, *Per capita* DALYs lost to VBPDs | *D = ln(365DALY s + 1).*The natural log of the sum of *per capita* disability-adjusted life years lost to: malaria, trypanosomiasis, Chagas disease, schistosomiasis, leishmaniasis, lymphatic filariasis, onchocerciasis, dengue, Japanese encephalitus, ascariasis, trichuriasis, and hookworm. Because the VBPD burden for some countries is 0 (which cannot be logged), the data were transformed by multiplying the DALYs by 365 and adding 1. This represents the number of days per year lost to VBPDs, with 1 day set as the minimum. Year: 2002. Data Source: [2]. |
| *I,* Institutions Index | *I=0.38VA+0.39PV +0.42GE+0.41RQ+0.43RL+0.41CC.* A composite index derived from principal components analysis of the following 2002 governance indicators: voice & accountability (VA), political stability & absence of violence (PV), government effectiveness (GE), regulatory quality (RQ), rule of law (RL), and corruption (CC). These indicators have been used to represent the quality of economic institutions in a number of other studies [3]. Data source: [4]. |
| *L,* Latitude | Official country latitude listed in the CIA World Factbook, 2007. |
| *T*, Tropics | Equals 0 if L is more than 23.5. Equals 1 otherwise. |
| *E*, Energy | *E = ln(O + G + C + 1).*The natural log of the *per capita* total value of oil (O), natural gas (G), and coal (C) production for 2002. A value of 1 is added to all countries, ensuring that the minimum logged value is 0. Data source: [5]. |
| *F*, Ethnolinguistic Fractionalization | The probability that two randomly chosen individuals from a given country are from different ethnic groups in 1985. This is frequently used as an IV in economic studies [6, 7]. Data source: [8]. |
| *K*, Landlocked | Equals 1 if the country has no coastline or the only coastlines are closed seas. Equals 0 otherwise. |
| *B*, Biodiversity | *B = 0.59mam + 0.61bir + 0.53pla*, where   $mam=\frac{MAM}{{Land}^{0.273}}$, $bir=\frac{BIR}{{Land}^{0.168}}$, and $pla=\frac{PLA}{{Land}^{0.279}}$.  This is a composite index derived from the first principal component of the density of mammal (mam), bird (bir), and plant (pla) species. Species densities were derived by estimating species area curves from OLS regressions of the natural log of total mammal (MAM), bird (BIR), and plant (PLA) species on the natural log of the total land area for each country (p = 0.001 for all estimates); R^2^ = 0.40, 0.30, and 0.24, respectively. Year: 2002. Data source: [9]. |
| Spatially-lagged dependent variables | Generated from spatial weights matrices, based on the Euclidean distance in latitude and longitude between countries. Code for calculating SLDVs in Stata 12 was provided by [10]. |
