# Supplementary material for: Disease Ecology, Biodiversity, and the Latitudinal Gradient in Income
Source: PLoS Biol. 2012 Dec 27;10(12):e1001456. doi: 10.1371/journal.pbio.1001456 (PMC3531233; doi:10.1371/journal.pbio.1001456)
Supplement: Table S2 — Analysis of tropical (a) and sub-tropical (b) countries. The parameter estimates are presented in columns a, b, and c. The corresponding independent variables are listed to their left. Robust standard errors are presented in parentheses below their corresponding parameter estimates. The lower sample size (and lower variability) among tropical countries results in few statistically significant estimates for the income equation. Nevertheless, the parameter estimates for the impact of disease on income is very similar across groups. IVInstrumented; lnnatural log; §units×10−2 units; ***significant at the 1% level; **significant at the 5% level; *significant at the 10% level. §units×10−2. (DOCX) [file pbio.1001456.s002.docx]

| **Table S2. Analysis of Tropical (a) and Sub-Tropical (b) Countries.** | | | | | | | |
| --- | --- | --- | --- | --- | --- | --- | --- |
| **Dependent Variable: Income** | | | | **Dependent Variable: *Disease*** | | | |
| **Independent Variables** | **Parameter Estimates** | | | **Independent Variables** | **Parameter Estimates** | | |
|  | **a. Tropics** | **b. Tropics + Subtropics** | **c. All** |  | **a. Tropics** | **b. Tropics + Subtropics** | **c. All** |
| **Disease^lnIV^** | -0.35 (0.64) | -0.31 (0.21) | **-0.40*** (0.09)** | **Income^ln^** | **-**0.29 (0.19) | **-0.30*** (0.10)** | **-0.16* (0.09)** |
| **Latitude^§^** | -0.62 (0.07) | 0.51 (1.32) | 0.24 (1.01) | **Latitude^§^** | **-5.39*** (1.53)** | **-4.26*** (1.23)** | **-2.99*** (0.81)** |
| **Landlocked** | -0.24 (0.64) | **-0.49* (0.14)** | **-0.54*** (0.21)** | **Biodiversity^§^** | **-0.31*** (0.08)** | **-0.26*** (0.05)** | **-0.29*** (0.05)** |
| **Energy^ln^** | **0.19** (0.09)** | **0.18*** (0.04)** | **0.12*** (0.03)** | **Island** | **-0.94** (0.41)** | **-0.62* (0.35)** | **-0.63*** (0.30)** |
| **Institutions^IV^** | 0.37 (1.31) | 0.40 (0.32) | **0.38*** (0.14)** | **Tropics** | **_ _ _ _ _** | **0.48* (0.27)** | **0.96*** (0.21)** |
| **Constant** | **7.61*** (1.17)** | **7.52*** (0.27)** | **7.79*** (0.26)** | **Constant** | **5.54*** (0.96)** | **4.79*** (0.69)** | **3.33*** (0.50)** |
| ***R^2^*** | 0.77 | 0.80 | 0.84 | ***R^2^*** | 0.67 | 0.73 | 0.76 |
| ***n*** | 69 | 100 | 139 | *n* | 69 | 100 | 139 |
