# Supplementary material for: Disease Ecology, Biodiversity, and the Latitudinal Gradient in Income
Source: PLoS Biol. 2012 Dec 27;10(12):e1001456. doi: 10.1371/journal.pbio.1001456 (PMC3531233; doi:10.1371/journal.pbio.1001456)
Supplement: Table S3 — First-stage results in the estimation of income equation (1) . Columns 2 and 3 represent parameter estimates for equations (9) and (10) respectively. The corresponding independent variables are listed on the left. Standard errors are presented in parentheses below their corresponding coefficient estimates; n = 139. ***Significant at the 1% level; **significant at the 5% level; *significant at the 10% level; §units×10−2 units. (DOCX) [file pbio.1001456.s003.docx]

| **Table S3. First-stage results in the estimation of income equation (1).** | | |
| --- | --- | --- |
| **Independent Variables** | **Dep. Variable: $\hat{D}$** | **Dep. Variable: $\hat{I}$** |
| **Latitude^§^** | **-3.94*** (0.60)** | **11.20*** (1.67)** |
| **Landlocked** | 0.14 (0.12) | **-1.09*** (0.37)** |
| **Island** | **-0.75** (0.31)** | **1.23** (0.58)** |
| **Tropics** | **0.95*** (0.24)** | 0.87 (0.58) |
| **Energy** | -0.03 (0.02) | -0.04 (0.06) |
| **Biodiversity^§^** | **-0.32*** (0.04)** | **0.29** (0.08)** |
| **Constant** | 2.52*** (0.32) | **-3.90*** (0.79)** |
| **R^2^** | 0.73 | 0.49 |
| Columns 2 and 3 represent parameter estimates for equation (9) and (10) respectively. The corresponding independent variables are listed on the left. Standard errors are presented in parentheses below their corresponding coefficient estimates; *n =* 139. ***Significant at the 1% level; **Significant at the 5% level; *Significant at the 10% level; ^§^ units x 10^-2^ units. | | |
