# Supplementary material for: Disease Ecology, Biodiversity, and the Latitudinal Gradient in Income
Source: PLoS Biol. 2012 Dec 27;10(12):e1001456. doi: 10.1371/journal.pbio.1001456 (PMC3531233; doi:10.1371/journal.pbio.1001456)
Supplement: Table S4 — First-stage results in the estimation of disease equation (2) . The right columns represent parameter estimates for equation (11). The corresponding independent variables are listed on the left. Standard errors are presented in parentheses below their corresponding coefficient estimates; n = 139. ***Significant at the 1% level; **significant at the 5% level; *significant at the 10% level; §units×10−2 units. (DOCX) [file pbio.1001456.s004.docx]

| **Table S4. First-stage results in the estimation of disease equation (2).** | |
| --- | --- |
| **Independent Variables** | **Dep. Variable: $\hat{M}$** |
| **Latitude^§^** | **6.02*** (1.04)** |
| **Landlocked** | **-1.01** (0.22)** |
| **Island** | **0.79** (0.35)** |
| **Tropics** | -0.06 (0.38) |
| **Energy** | **0.11*** (0.04)** |
| **Biodiversity^§^** | **0.19*** (0.05)** |
| **Constant** | **5.33*** (0.50)** |
| **R^2^** | 0.58 |
