# Supplementary material for: Disease Ecology, Biodiversity, and the Latitudinal Gradient in Income
Source: PLoS Biol. 2012 Dec 27;10(12):e1001456. doi: 10.1371/journal.pbio.1001456 (PMC3531233; doi:10.1371/journal.pbio.1001456)
Supplement: Text S1 — Supplementary information: variable definitions and data sources; assumptions and limitations of the analysis; supplementary analyses. (DOCX) [file pbio.1001456.s005.docx]

**SUPPORTING INFORMATION**

**APPENDIX**

***Variable Definitions and Data Sources***

Additional information on the variables used in the analyses is available below in Table S1.

Countries included in the analysis are: Albania, Algeria, Angola, Argentina, Armenia, Australia, Austria, Azerbaijan, Bangladesh, Belarus, Belgium, Benin, Bhutan, Bolivia, Botswana, Brazil, Bulgaria, Burkina Faso, Burundi, Cameroon, Canada, Central African Republic, Chad, Chile, China, Colombia, Congo Brazzaville, DRC, Costa Rica, Cote d'Ivoire, Croatia, Czech Republic, Denmark, Dominican Republic, Ecuador, Egypt, El Salvador, Equatorial Guinea, Estonia, Ethiopia, Fiji, Finland, France, Gabon, Gambia, Georgia, Germany, Ghana, Greece, Guatemala, Guinea, Guinea-Bissau, Guyana, Haiti, Honduras, Hungary, Iceland, India, Indonesia, Iran (Islamic Republic of), Ireland, Israel, Italy, Jamaica, Japan, Jordan, Kazakhstan, Kenya, South Korea, Kyrgyzstan, Lao People's Democratic Republic, Latvia, Lebanon, Lesotho, Liberia, Libyan Arab Jamahiriya, Lithuania, Macedonia, Madagascar, Malawi, Malaysia, Mali, Mauritania, Mexico, Moldova, Mongolia, Morocco, Mozambique, Namibia, Nepal, Netherlands, New Zealand, Nicaragua, Niger, Nigeria, Norway, Oman, Pakistan, Panama, Papua New Guinea, Paraguay, Peru, Philippines, Poland, Portugal, Romania, Russian Federation, Rwanda, Saudi Arabia, Senegal, Sierra Leone, Slovakia, Slovenia, Solomon Islands, South Africa, Spain, Sri Lanka, Sudan, Suriname, Sweden, Switzerland, Syrian Arab Republic, Tajikistan, Tanzania, Thailand, Togo, Tunisia, Turkey, Uganda, Ukraine, United Kingdom, United States of America, Uruguay, Uzbekistan, Venezuela (Bolivarian Republic of), Vietnam, Yemen, Zambia, Zimbabwe.

***Assumptions and Limitations***

Below we address limitations of three aspects of the analysis: the model, IV methods, and data.

*Limitations of the Linear Model*

The statistical model used in this study is a regression model that is linear in the parameters. In the economics literature, this approach is standard, which may be most appropriate when the relationship between the explanatory variables and the dependent variable is expected to be monotonic (though non-monotonic functions can also be estimated) and can be linearized through simple methods such as natural logs. A log-linear structure is intuitive for the relationship between health and income for which theory and evidence are in agreement about the expected effects [11,12]

One of the premises of this study is that the distribution of vector-borne and parasitic diseases is determined by ecological processes. A fundamental difference between economic and ecological perspectives on health is the consideration of underlying complexity that influences the disease burden. This is especially true for the effect of biodiversity on VBPDs, which we know is influenced by a range of complex processes related to predation, competition, and population genetics that may be nonlinear, and may vary considerably between diseases. Our statistical model only tests the relationship between aggregate values of VBPDs and biodiversity, for which no general theory exists. Our model therefore aims only to understand general trends, which we measure empirically.

To ensure that the underlying complexity of biodiversity was not distorting our results, we considered two other variations of the model that were not presented in the main text. The first included a quadratic term for biodiversity in the disease equation (i.e., ${(\mu}_{1}B+\mu_{2}B^{2}$) and the second excluded biodiversity altogether, using islands as the IVs for disease. Our general results are robust to both variations of the model. The parameter estimate for $B^{2}$ was statistically significant but ecologically trivial $(\mu_{2}=0.000002)$, and had no significant effect on other parameters of interest. The main conclusions were also preserved when we removed biodiversity entirely from the analysis; the estimated effect of disease on income (without biodiversity as an instrument) was *-0.42* (*p*= 0.05), which is in the middle of the range of results presented in Table 4. Another potential source of nonlinearity could be geography: the relationship between disease and income (or between biodiversity and disease), could be fundamentally different in tropical versus temperate regions. The dummy variable for tropical countries only controls for a linear affect of tropical conditions, but does not allow for the slope of other factors to change. To address this issue, we tested our model for only tropical and subtropical countries, with the results presented in Table 7 below. The disease equation generated almost identical results, with an estimated effect of biodiversity on disease for tropical countries of *-0.31* compared to *-0.29* for all countries (*p=0.01*). The parameter estimates for the income equation were also very similar: the estimated effect of disease on income was -0.35 for tropical countries compared to *-0.40* for all countries. However, because of low sample size (*n=69*) and relatively low variability of income, most of the parameters were not statistically significant in the income equation for tropical countries. These common parameter estimates do not indicate that a fundamental shift occurs for only tropical countries, and they provide some support for controlling for tropical countries through a dummy variable, as we do in the main analysis.

Beyond nonlinearities, there is a range of other variables that were considered. Among exogenous variables that have been suggested to be relevant are temperature and rainfall. Neither of these were found to be independently significant. A dummy variable for tropical countries was also considered in the income equation, but was invariably statistically insignificant. Other variables that have been suggested are education levels, capital stocks, and population density, which are endogenous to the system (i.e., they are affected by income), and are disqualified under the excludability criterion. Nevertheless, we considered additional analysis with population density as a control variable, which was insignificant for both the disease and income equations, with the results for other parameters barely affected. We also considered a specification with education as a component of the institutions equation, which generated very similar results as the model without it. Finally, the high *R^2^* (which ranges between *0.60* and *0.87* for all models) further supports that our simple linear model does an effective job of fitting the data.

*Assumptions and Limitations of Instrumental Variables*

The normal set of assumptions for linear regressions apply to the second-stage of a two-stage regression analysis. The IV is used specifically for the purposes of overcoming simultaneity bias when an explanatory variable is endogenous. The basic requirements for the IVs are 1) that they are correlated with the endogenous explanatory variable, and 2) that they are independent of the dependent variable; i.e., that they are uncorrelated with the error term [13]. The first issue is straightforward because the correlations between the IVs and the endogenous variables are tested. Island and biodiversity are empirically strong instruments. A range of plausible alternative control variables were also tested in the model to account for potential omitted variable bias. The second issue is a more serious concern because it is not fully testable [13]: exclusion restrictions require that IVs be excluded from the second-stage regression. If the model has exactly as many IVs as endogenous variables, then it is said to be “just identified”, and IVs cannot be used in the second stage regression (their excludability cannot be tested). However, if the model has more IVs than endogenous variables, then it is considered “over-identified” and the test of over-identifying restrictions provide an indication of whether the IVs are correlated with the error term. This test assumes that at least one of the IVs is truly exogenous. We present these identification tests in Tables 4 and 5.

Our main conclusions would be compromised if islands or biodiversity were independently correlated with *per capita* income in ways that are not controlled for by other variables. This is unlikely in our system. From an economic perspective, island countries are distinguished by two features: ports and size. We control for ports in our system via a dummy variable for landlocked countries. In our case, size is not a factor because most small island countries were excluded due to lack of complete data. The islands countries that we consider represent a range of sizes, histories, and latitudes: Australia, Fiji, Iceland, Jamaica, Japan, Madagascar, New Zealand, Philippines, Solomon Islands, and Sri Lanka.

Intuitively, one might expect that there could be a bias between biodiversity and disease through indirect correlations with income: i.e., economic growth is associated with less biodiversity (due to loss of habitat), which is in turn associated with higher living standards and less disease. The reason why the biodiversity index is unlikely to have been influenced by economic development is because it refers strictly to the density of different species (not abundance) of plants, mammals, and birds found within each country (for more details see Table 6). This index is mostly driven by plant species richness, which is not generally sensitive to economic development [14, 15].

*Limitations of the Data*

The data are collected from a range of publicly available on-line sources described in Table 6. The final estimations are from a single cross section for all 139 countries for which we had complete data for 2002. The year 2002 is the only year for which we have a data set for the burden of VBPDs, which precludes incorporating multiple years into the analysis. There is an implicit temporal component that is based on the fact that differences in income between countries are due to differences in economic growth rates over the course of history. Because the global economy has experienced divergence over the past two hundred years, analyses conducted on earlier data (e.g., eighty years ago) would presumably generate lower parameters estimates of the income equation. Likewise, analyses conducted on data fifty years from now would presumably generate larger parameter estimates.

There were no outliers in the final data set (the fitted values are presented in Figure 3), but there may be outlier countries that were not included for lack of data (such as Singapore). Of all of the variables, the one with the most uncertainty is the disease burden, which represents the official statistics of the World Health Organization, but is estimated from a broad range of sources. Two potential concerns regarding data accuracy are uncertainty and bias. Unreliable data can create large standard errors, making statistical inference more difficult. In our study, the estimates are highly significant, so variation in the reliability of the data is only a problem if it is biased. We have no reason to believe that the data on disease burdens are biased.

Of the whole analysis, the result that is most surprising is the consistency with which biodiversity predicts lower burdens of VBPDs. The biodiversity index is based on species densities of plants, birds, and mammals. We estimated species area curves for each taxon, where the natural log of species richness is regressed against the natural log of land area for each country. The index is then a linear combination of species densities of the three taxa (details are in Table 6). This is the most straightforward way to generate a species richness index. However, it is nevertheless possible that there is underlying nonlinearity, or spurious correlations, between species richness and disease that is a result of the construction of the index that has eluded detection in the analysis. Further studies should probe deeper into these relationships.

***Spatial Autocorrelation***

An additional relevant factor in this analysis is spatial dependency (or autocorrelation). There are two potential sources of spatial autocorrelation: 1) direct effects (i.e., *D_j_ = D_j_(D_j-1_)* where *j* represents a point in space, and 2) spurious effects (i.e., *D_j_ = D_j_(X_j_(X_j-1_))* and *D_j-1_ = D_j-1_(X_j-1_(X_j_))* where *X* is an explanatory variable). Spatial dependency is a common consideration in biology because the distribution of organisms is usually affected directly by spatial mechanisms; i.e., infectious diseases transmit from one geographic neighbor to the next through a biological mechanism. Economists, however, do not typically control for space in macro-level studies because geographic proximity is often not considered fundamental to international trade. However, there are many reasons to believe that spurious spatial dependency is relevant in both biology (e.g., the formation of common habitats) and economics (spatially-dependent histories due to patterns of regional politics, immigration, or conflict).

We use two tests for spatial autocorrelation. For preliminary analysis of raw data, we use Moran's I tests on spatially-lagged variables that are generated from spatial weights matrices, based on the Euclidean distance in latitude and longitude between countries (Stata code for these methods can be found at [16,10]. Both the income data (Moran's *I, p = 0.00*) and the disease data indicated statistically significant spatial autocorrelation (Moran's *I, p = 0.00*). However, this preliminary evidence of spatial autocorrelation does not necessarily imply that geographic proximity is a fundamental driver; as mentioned above, there may be spurious drivers such as latitude or tropical conditions.

A more appropriate test for spatial autocorrelation is therefore in the residuals of the models after the parameters are estimated (i.e., after controlling for confounders) in the tradition of [17] (Stata code can be found at [18]. There was no statistically significant evidence of spatial autocorrelation in either income (IV Moran’s, p = 0.58) or disease (IV Moran’s, p = 0.11) in the full model (that included latitude and tropics). However, there was evidence of spatial autocorrelation for a subset of the models. As an extra precaution, to ensure that spatial processes have not confounded our results, we explicitly control for spatial autocorrelation in two estimations. Model 7 (Tables 4 and 5, Column 7) explicitly controls for spatially-lagged dependent variables (SLDVs), which adds a layer of complexity but does not generate statistically different results.

Controlling for SLDVs presents a new source of endogeneity because the disease burden of country *i* and the distance-weighted disease burden of its neighbors influence each other. To overcome this, the SLDVs must therefore also be instrumented from first-stage regressions [19,20]. By convention, the IVs for the SLDV are spatially lagged independent variables (SLIVs). Finally, as an alternative way for controlling for space, in model 8 (Tables 4 and 5, Column 8), we included dummy variables for sub/continents. Because our limited observations preclude simultaneously including all sub/continents in the analysis, we selected sub/continents via a stepwise process, where sub/continents that were not statistically significant at the 10% level were iteratively excluded. Our conclusions were robust to every one of these different model specifications.

***Generating Figure 4***

Figure 4b is a partial correlation plot of the biodiversity index on the burden of VBPDs. It represents the estimated correlation of biodiversity and the burden of VBPDs after controlling for the influence of other variables. Specifically, “Biodiversity|Others" are the residuals from a regression of biodiversity, *B*, on all other variables in the disease equation (8):

| $B=f_{6}(\hat{M},L,T,S)$ | (12) |
| --- | --- |

where $\hat{M}$ represents the predicted values from a first-stage regression of income on all exogenous variables, except biodiversity:

| $\hat{M}=f_{7}(L,T,K,E,S)$ | (13) |
| --- | --- |

“VBPD|Others" are the residuals from a regression of the VBPD burden, *D*, on all other variables in the VBPD equation (8), except for biodiversity:

| $D=f_{8}(\hat{M},L,T,S)$ | (14) |
| --- | --- |

***First Stage Regressions***

The first stage regressions are presents Tables S8 and S9.

**SUPPORTING REFERENCES**

1.  World Bank Development Data Group (2007) 2007 World Development Indicators Online, http://go.worldbank.org/3JU2HA60D0, The World Bank, Washington, DC.

2. Lopez AD, Mather CD, Ezzati M, Jamison DT, Murray CJL (2006) Global Burden of Disease and Risk Factors. (D.C.): World Bank.

3.  Easterly W, Levine R (2003) Tropics, germs, and crops: How endowments influence economic development. J Mon Econ 50: 3–39.

4.  Kaufmann D, Kraay A, and Mastruzzi M (2005) Governance matters IV: governance indicators for 1996-2004. Policy Research Working Paper Series. The World Bank (3630).

5  BP p.l.c. (2007) Statistical Review of World Energy, BP p.l.c., London, UK Available online at http://www.bp.com/statisticalreview.

6.  Mauro P (1995) Corruption and growth. Q J Econ 110: 681–712.

7.  Easterly W, Levine R (1997) Africa’s growth tragedy: Policies and ethnic divisions. Q J Econ 112: 1203–1250.

8.  Roeder PG (2001) Ethnolignuistic fractionalization (ELF) indices, 1965 and 1985 http//:weber.ucsd.edup ̃roeder, 2001.

9.  World Conservation Monitoring Center (2002) UNEP-WCMC Species Database, UNEP-WCMC, Cambridge.

10. Jeanty PW (2010) splagvar: Stata module to generate spatially lagged variables, construct the Moran scatter plot, and calculate global Moran’s I statistics. Available from http://ideas.repec.org/c/boc/bocode/s457112.html.

11. Bloom D, Canning D, and Sevilla J. (2003) Geography and poverty traps. J Econ Growth 8: 355–78.

12. Weil DN (2007) Accounting for the effects of health on economic growth. Q J Econ 122: 1265–1306.

13. Murray M (2006) Avoiding invalid instruments and coping with weak instruments. J Econ Perspec 20: 111–132.

14. Honnay O, Hermy M, Coppin P (1999) Effects of area, age and diversity of forest patches in Belgium on plant species richness, and implications for conservation and reforestation. Biol Cons 87: 73–84.

15. Helm A, Hanksi I, Partel M (2006) Slow response of plant species richness to habitat fragmentation. Ecology Letters 9: 72–77.

16. Jeanty PW (2010) spwmatrix: Stata module to generate, import, and export spatial weights. Available from http://ideas.repec.org/c/boc/bocode/s457111.html.

17.  Anselin L (2007) Testing for spatial autocorrelation in the presence of endogenous regressor. Inter Reg Sci Rev 20: 901–969.

18. Jeanty, P W (2010) anketest: Stata module to perform diagnostic tests for spatial autocorrelation in the residuals from OLS, SAR, IV, and IV-SAR models. Available from ideas.repec.org/c/boc/bocode/s457113.html.

19.  Anselin, L (2007) Spatial econometrics In T C. Mills and K. Patterson, (eds.), Handbook of Econometrics, pp. 901–969 Palgrave MacMillan.

20.  Fingleton B, Le Gallo J. (2008) stimulating spatial models with endogenous variables, a spatial lag and spatially dependent disturbances: Finite sample properties. Papers Reg Sci 87: 319–339.
